# The Inspection of Meat

**Published:** 1912-04-27

**Authors:** 


### THE INSPECTION OF MEAT.

### The Present System and Where it Needs Reform.

The inspection of meat is comparatively a new thing among Christian nations, though it has been controlled by law for centuries past by Jew and Mussulman. The latter concerns himself entirely with a ritual that any true believer is competent to perform, and asks only that sufficient ceremony has been used to make the meat lawful; the former goes deeper, and sees to it that the meat is not only lawful by ceremonial slaughter, but certainly wholesome. The Mussulman is quite satisfied if before a beast is quite dead its throat has been cut by a co-religionist with ceremonial invocation of Allah, and if blood has flowed. Amongst the lower classes of the faithful in India the slightest quiver of the body is evidence of life, the feeblest trickle is a flow of blood. This ceremonial they must have to make meat lawful with them. Even if but feeble protection, this results in an inspection that guards against the eating of a beast that has died of disease.

The Jewish laws are public health regulations. The Kosher meat killed with ceremony is inspected for, and rejected for, disease. To Jew and Mussulman certain species are unlawful. The foundation of this regulation was the unclean feeding of the unlawful animals.

The Christian, because he asks no ceremonial in the killing, and because, though prejudiced against eating some, he considers no species unlawful, till comparatively lately did not concern himself with inspection. So long as disease had not left visible trace on the joint none asked questions, till bacteriologists opened all eyes to possible dangers. Even now it is not unusual in country districts to "kill a beast to save its life," as the saying is, and pass the meat on at a low price to the shop of an unscrupulous butcher. Round every sale-ring there are men waiting to buy "screws," often tuberculous beasts, for a few pounds or shillings. What becomes of these?

In the large towns meat inspection is an important duty, sometimes the only duty, of a highly trained or much-experienced man, but in smaller towns and in villages, if there be any inspection, the duty devolves on petty officials, such as inspectors of nuisances and policemen. The former may be little instructed, and frequently are local men more inclined to close their eyes than by condemnation to involve a life-long neighbour in a loss; the policeman seldom has sufficient knowledge to enable him to condemn anything that does not stink.

In Southern England it is the custom in many villages, when a porker is fit to die, for its owner to send round amongst his neighbours to inquire how many will take a piece. If the subscribers are sufficient, the porker's fate is sealed, but if the demand be small he may live to be bacon. Many sheep are killed on like terms by farmers, who sell the mutton locally at a good profit for less than the butcher demands. In this way the cottagers get more meat than they could afford at shop prices. Had all beasts to go to a public slaughter-house and to be seen by the inspector it would necessitate in each village the settling of a day and an hour in each week or fortnight for the visit of an inspector common to several villages. This would not fall in with such arrangements as have been mentioned. It is easier to legislate than to foresee the effects of the law.

The Royal Sanitary Institute has issued a syllabus of a course of some forty lectures and demonstrations that in a little over a month is to fit any man over the age of one-and-twenty to fulfil all the duties of an inspector of food—"to cover the whole field of food inspection," to quote the circular. The Local Government Board, with that touching faith in the efficacy of lectures and the sufficiency of the hall-mark of successful passing of examinations that distinguishes the Government Departments of China and Great Britain, has accepted the examination as sufficient proof of knowledge.

That any man in the time allowed can learn so thoroughly as to be efficient in such a tricky subject as food inspection must be doubted, though it cannot be doubted that active brains can cram enough in the time to scrape through the examination.

That most needful for real knowledge of the condition of food-stuff is constant practice in judging it. The object of this course of lectures appears to be to enable men to get certificated without long apprenticeship. It is as if the Colleges of Surgeons and Physicians allowed a man to qualify to practise after producing evidence of six months' cramming. To put a final polish on the men who for a year have worked under an experienced inspector in some large town, this course would be hard to better; but to let it be a means of avoiding drudgery in a subordinate position is a mistake. But that it is intended to make easy and rapid the acquisition of sufficient knowledge to get a certificate the circular itself is evidence, for it says openly: "Evidence of having attended the following courses of lectures and demonstrations will be accepted by the Committee in lieu of the practical training required by the regulations. The Royal Sanitary Institute," etc., etc. There is the mistake, "in lieu of the practical training." There is no royal road to learning. Rather should it be "in addition to the practical training."

Without practical training, and plenty of it, no man will be an efficient inspector. The examination for certificate should come after practical experience has been gained, not before setting forth to acquire it, or the certificate will be cheapened and the certificated inspector will be an easy prey to the astute rascals it is his business to keep in check. Be it remembered, it is to guard the consumer from men up to every move in the game of foisting bad food on to the public that the inspector exists. A year of practical training, then such a course as the syllabus sets forth, and a man should be a valuable servant of the public and worthy of his wage.

